# Organizational injustice and sickness absence: The moderating role of locked-in status

**DOI:** 10.1016/j.ssmph.2023.101427

**Published:** 2023-05-13

**Authors:** Paraskevi Peristera, Johanna Stengård, Constanze Eib, Claudia Bernhard-Oettel, Constanze Leineweber

**Affiliations:** a Department of Psychology, Stockholm University, Stockholm, Sweden; b Department of Psychology, Uppsala University, Uppsala, Sweden

**Keywords:** Organizational overall (in)justice, Frequent and short sickness absence, Duration of sickness absence, Locked-in status, Longitudinal SEM models

## Abstract

Organizational injustice is known to negatively affect employees’ health and to increase the risk for sickness absence. The negative health effects are also known to be more pronounced in uncontrollable, strain increasing, situations at the workplace. This study tests whether locked-in status, i.e., being stuck in a non-preferred workplace, modifies the associations between injustice perceptions and frequent (≥2 times/yr) and long (≥ 8 days/yr) sickness absence. The sample contained 2631 permanent employees from the Swedish Longitudinal Occupational Survey of Health in 2018 and 2020. Multigroup structural equation modelling was used to compare the proposed relationships between employees who are locked-in in their workplace and employees who are not. We found a positive association between higher overall organizational injustice and long sickness absence two years later, with the association being stronger for the locked-in group. Also, higher injustice was associated with more frequent sickness absence, but only for those not being locked-in.

Employees being locked-in seem to have higher risk of long-term sickness absence which might indicate more serious health problems. Employees not being locked-in more often take short sickness absence, which could indicate a coping behaviour to handle high strain. This study adds knowledge to the role of locked-in status as a moderator in the much-studied relationship between organizational justice and health as well as to the multiple reasons underlying sickness absence.

## Highlights

• High organizational injustice is associated with longer sickness absence both for locked-in and not locked-in employees.
• Individuals that are locked-in may be more strongly affected by destructive working factors such as organizational injustice.
• Examination of the moderating role of locked-in status, clarifies how different workplace stressors interact.
• For employees being locked-in, organizational injustice is related to longer sickness absence impying delay return to work.
• Organizations should improve employability and increase justice at work so as to reduce sickness absence.

## 1 Introduction

Organizational justice is known to affect employees’ health and well-being. Previous studies examining different dimensions of justice, i.e., distributive, procedural, and interactional justice (Colquitt, 2001; Colquitt et al., 2005) found longitudinal associations with stress reactions, depressive symptoms and sleeping problems (Bernhard-Oettel et al., 2019) (Bernhard-Oettel et al., 2019), emotional exhaustion (Clements & Kinman, 2021), pain(McParland et al., 2022) and self-rated health (Bernhard-Oettel et al., 2019; Clements & Kinman, 2021; Kivimaki et al., 2003; Leineweber et al., 2016; McParland et al., 2022) as well as with sickness absence (Leineweber et al., 2017; Tenhiälä et al., 2013; Ybema et al., 2016).

Moreover, there is longitudinal evidence that distributive justice (Leineweber et al., 2017; Ybema & van den Bos, 2010) and procedural justice contribute to lower sickness absence (Eib, 2018). Similar results from a Finnish study showed that procedural and interactional justice are associated with subsequent sickness absence (Elovainio et al., 2013). Similarly, Tenhiälä (2013) showed that older workers who experienced high levels of procedural justice were less likely to miss work due to medically certified illnesses (Tenhiälä et al., 2013). To summarize, several longitudinal studies using objective assessments of sickness absence found support for an association between various justice indicators and sickness absence (Hjarsbech et al., 2014; Väänänen et al., 2004). Furthermore, self-reported shorter sickness absence was found to relate to motivation and commitment (Boer et al., 2002; Johansson, 2002). Although sickness absence cannot be considered to directly measure health it is strongly related to it. Research has shown associations between ill-health (e.g. self-rated health, depressive symptoms, burnout) and sickness absence (Koopmans et al., 2008; Peterson et al., 2011; Taimela et al., 2007).

In contrast to separate dimensions of justice, overall justice captures the extent to which employees perceive justice from their organization as a whole; a conceptualization that may reflect more accurately and parsimoniously how employees experience fairness at their workplace (Ambrose et al., 2015). In this study, we examine overall injustice and its association to sickness absence.

Empirical evidence shows that health effects related to unfair treatment at work tend to be stronger when people are confronted with uncontrollable or stressful situations, such as lack of work control, negative changes at work, organization's economic instability, experience of major life events or environmental stressors outside work (Elovainio et al., 2005; Hjarsbech et al., 2014; Peutere et al., 2019). Another situation that may increase the extent of feeling out of control is being locked-in in a workplace.

The concept of “workplace locked-in” has originally been operationalized as being in a non-preferred occupation or an undesired workplace (Aronsson et al., 2000; Aronsson & Göransson, 1999). A later operationalization suggests adding perceived employability to the previous definition (Fahlén et al., 2009; Stengård et al., 2016). In this study, we adopt this latter operationalization according to which employees are considered as being workplace locked-in when they (a) have a low preference for remaining in their workplace in the future and also (b) perceive low possibility of finding another equivalent job with a different employer.

Previous research has shown that being workplace locked-in affects employees’ health and well-being (Aronsson & Göransson, 1999; Furåker & Saloniemi, 2014; Stengård et al., 2016). For instance, locked-in employees report more often headaches, fatigue, upper-back pain, stomach aches, spinal pain, sleeping problems (Aronsson et al., 2000; Aronsson & Göransson, 1999; Furåker & Saloniemi, 2014; Muhonen, 2010) as well as worse mental health (Canivet et al., 2017; Muhonen, 2010; Skillgate et al., 2021; Stengård et al., 2016). Research examining the association between being workplace locked-in and sickness absent is scarce. There is only one cross-sectional study showing that being workplace locked-in is associated with long-term sickness absence (Fahlén et al., 2009). Yet, it has not been explicitly examined if the association between overall injustice and sickness absence is modified by the workplace locked-in status of employees.

It may be the case that workplace locked-in employees are particularly vulnerable when they are exposed to detrimental working factors such as organisational injustice. If people experience injustice at work, and feel also locked-in, they are in an unpredictable situation (injustice) that they have low control over (workplace locked-in), and thus, cannot leave. This may raise their perceived strain. As locked-in employees cannot find a comparable job elsewhere, they are forced to endure their strain inducing situation. As both injustice and workplace locked-in are known to relate to ill-health, we expected injustice to have a more detrimental effect on health and sickness absence among locked-in employees.

Sickness absence is a complex phenomenon, which can be the consequence of ill-health (Marmot et al., 1995), as well as a coping mechanism (Kristensen, 1991). The need for considering different measures of sickness absence has been previously discussed (Borritz et al., 2006; Leineweber et al., 2017; Stengård et al., 2017). Here, we focus on two aspects of sickness absence and investigate whether relationships exist between organizational injustice, workplace locked-in status and frequent but short (high frequency, short duration), or infrequent but long-term leaves (low frequency, long duration). Long sickness absence duration and more severe sickness were expected for individuals locked-in a workplace while experiencing injustice, since they have probably endured a harmful situation for a longer period of time. More frequent sick leave could be used as a coping mechanism to get relief from a poor workplace and to recover from high strain. In addition, individuals with low employability may avoid taking sick-leave due to bears of being dismissed. Indeed, studies indicated that people are less likely to take sick leave when they are faced with the potential threat of job loss and unemployment (Hansen & Andersen, 2008; Heponiemi et al., 2010). If this is the case, those who are not in a workplace locked-in status may be more likely to frequently use sick leaves. Instead, workplace locked-in is a phenomenon more often seen among permanent workers, who have been in their workplaces for a long time and feel relatively secure. A pattern of frequent but short sickness absences may be a way of escaping; in that case we would expect similar effects as for duration, i.e., a stronger association among those feeling locked-in.

This study aims to investigate whether employees’ workplace locked-in status (employees being locked-in vs. not being locked-in) modifies the association between overall injustice and sickness absence with respect to frequency and duration.

## 2 Methods

### 2.1 Study population

Data from the Swedish Longitudinal Occupational Survey of Health (SLOSH), an approximately representative sample of the Swedish working population (Magnusson Hanson et al., 2018), was used. Since its inception in 2006, SLOSH has been conducted biennially and it comprises all participants from the 2003 to 2011 Swedish Work Environment Survey (SWES) (n = 40,877). SWES, which is carried-out by Statistics Sweden is a biennial self-completion questionnaire survey about the physical and psychosocial work environment, answered by a stratified selection of respondents to the Labor Force Survey (LFS). At each wave, respondents completed one of two questionnaires: one for people in paid work for ≥30% of full-time and another for people working <30% or who have left the labor force. The questionnaires are divided into three sections. The first section focuses on several aspects of the psychical and psychosocial work environment. The second section relates to health, while the third one concerns socio-demographic and economic conditions, including social factors outside work and more general aspects of wellbeing and quality of life.

**Fig. 1:**
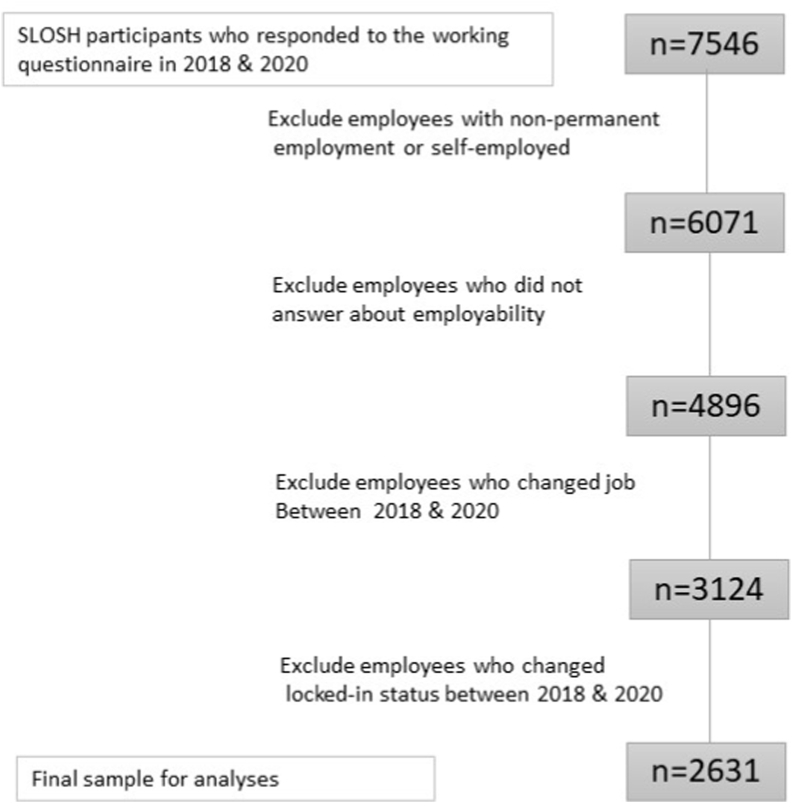
Flowchart of the data sample.

Our initial sample consisted of 7546 individuals who have responded to the working questionnaire of SLOSH both in 2018 and 2020. We restricted it to participants permanently employed in both years (n = 6071) as the meaning of being workplace locked-in presumably differs between temporary and permanent workers (Stengård et al., 2016). Previous research has acknowledged that even though temporary workers in some circumstances might be considered locked-in, it is questionable whether their situation accurately reflects characteristics of the locked-in phenomenon, since their situation may change whenever their contract ends (Furåker, 2010). To assess the condition of low employability in the locked-in definition, we excluded 4896 individuals who answered “I do not know” when asked whether they could easily find another equivalent job (Stengård et al., 2016). To avoid changes in organizational justice and/or locked-in status being due to job changes, the sample was restricted to respondents who worked in the same organizational setting during the study period. Thus, we excluded individuals who had changed job at least once between 2018 and 2020, ending up with 3124 individuals. Further, to be able to form groups according to workplace locked-in status, 493 employees who changed workplace locked-in status over time were excluded. Our final sample consisted of 2631 employees, with 2311 employees not being workplace locked-in neither year and 320 employees being workplace locked-in at both times (see Fig. 1). The Regional Research Ethics Board in Stockholm approved the study (Dnr: number omitted for peer review).

Dropout analyses were performed to examine potential differences between those individuals excluded from our analyses (n = 4915) and those who were included (n = 2631). We found no statistically significant differences in terms of gender (for women: 57.96% vs. 59.76%, p = 0.119) or long sick leave between individuals who were excluded and those who were included in the analyses (20.65% vs. 22.33%. p = 0.081). However, we did find differences in terms of age, locked-in status, overall injustice, frequent sick-leave and self-rated health. Excluded individuals (M = 51.57, SD = 9.2) were in average significantly younger than those included (M = 52.70, SD = 7.9; p < 0.001). We also found that excluded individuals were less often locked-in (13.76%) as compared to individuals included in the analysis (18.01%, p < 0.001). Further, individuals who remained in the study sample reported somewhat higher levels of injustice (M = 2.72, SD = 1.2) as compared to those excluded (M = 2.64, SD = 1.2; p < 0.05). Finally, we found that the average self-rated health for excluded individuals (20.17%) was significantly lower than for individuals in the analytical sample (22.93%, p < 0.001). Also, those included in the analytical sample were more often on frequent sick leave (22.93%) compared to those excluded (20.17%; p < 0.001).

### 2.2 Measures

Frequency of sickness absence was based on a question ‘How often have you taken sick leave for a week or less in the past 12 months?’ with response options 1 = 'never’, 2 = 'once’, 3 = 'two or three times', 4 = 'four times or more’. In line with previous studies, respondents were defined as being on frequent sickness absence if they had taken sick leave twice or more during the past 12 months (Leineweber et al., 2012). Duration of sickness absence was measured by one question ‘For roughly how many days in total have you been on sick leave during the past 12 months?’ with response options 1 = ‘Never’, 2 = ‘1–7 days', 3 = ‘8–30 days', 4 = ‘31–90 days', and 5 = ‘91 days or more’. In Sweden, employees need a doctor's certificate if not returning to work after seven days of sickness absence. For this reason, we created a dichotomous variable for duration of sickness absence, defined as 1 = long sickness absence (indicating sick leave of 8–30 days or more during the past 12 months) and 0 = short sick leave (7 days or less).

Overall organizational injustice was measured by six items following the sentence: “In this organization …“. For example, one of the items is “overall, I am treated fairly” (Ambrose et al., 2015). The items were scored on a Likert scale from 1 = strongly disagree to 7 = strongly agree. Responses to four of the items were reversed, such that higher values indicated higher levels of organizational injustice. The Cronbach's alpha was α = 0.90 in both waves, which is above the conventionally expected level of 0.70, indicating a satisfactory internal consistency. Overall injustice was fitted as a latent variable comprising six indicators.

The workplace locked-in status variable was created by combining questions about perceived employability and extent of job preference (being or not in a preferred job). Employability was based on the question “How easy would it be for you to get another, comparable, job without having to move?” with response options: 1 = very easy, 2 = rather easy, 3 = rather hard, 4 = very hard, and 5 = I don't know. Participants who answered the response alternative “5 = 1 don't know” were excluded from the analyses, in order to assess the condition of low employability in the locked-in definition (Stengård et al., 2016). Employability was coded as a dichotomous variable with 0 = indicating high employability (very/rather easy), and 1 = indicating low employability (rather/very hard). Job preference was measured with the question “Is the company/workplace where you work today the place you wish to work at in the future?“(Aronsson et al., 2000; Aronsson & Göransson, 1999), with three response alternatives: 1 = Yes, 2 = No, but I'm satisfied right now, 3 = No, I'm not satisfied with the workplace. The dichotomous variable workplace locked-in status was defined as 1 = being locked in a (comprising employees who were unsatisfied with their job and perceived their own employability as low as well as with employees who were only momentarily satisfied with their workplace for the momentand also perceived their own employability as low) and 0 = not being locked-in in a workplace (all other).

Register based sociodemographic variables (i.e., gender (0 = men, 1 = women), age (0=<35, 1 = 35–44, 2 = 45–54, 3 = 55–64, 4 = 65+ years), and socio-economic status (1 = unskilled workers, 2 = skilled workers, 3 = assistant non-manual workers, 4 = intermediate non-manual workers, 5 = professionals, higher civil servants)) were included in the analysis. In addition, the influence of a potentially confounding variable, which controlled for whether a change of manager occurred during the study period (0 = yes, 1 = no) was assessed, since due to changing manager having been shown to potentially affect employees’ perceived justice (Fortin et al., 2016; Jones & Skarlicki, 2013).

### 2.3 Analytic strategy

All analyses were performed in Mplus 8.4. (Muthén & Muthén, 1998). First, we tested measurement invariance over time and across workplace locked-in and not-locked-in subgroups for overall injustice. We used confirmatory factor analysis (CFA) in a structural equation modelling (SEM) framework (Putnick & Bornstein, 2016) by comparing a series of nested models: 1) a configural invariance model, 2) a metric invariance model, 3) a strong invariance model, and 4) a strict invariance model.

Multigroup structural equation analysis was then applied to test group differences by locked-in status (Asparouhov & Muthen, 2012). More specifically, autoregressive and cross-lagged effects between a) overall injustice and frequency of sickness absence, and b) overall injustice and duration of sickness absence, were estimated at each time point in a model stratified by locked-in status. The cross-lagged paths estimated the effect of one variable on the other between 2018 and 2020. Each path in the models was adjusted for gender, age, socio-economic status and change of manager.

Model fit indices include the comparative fit index (CFI), the Tucker-Lewis index (TLI), the standardized root mean squared of residuals (SRMR), and the root mean square error of approximation (RMSEA), where values of RMSEA ≤0.06, SRMR ≤0.08, CFI and TLI ≥0.95 indicated good fit (Hu & Bentler, 1999). Due to the more commonly used chi-square difference test being sensitive to minor parameter changes in large samples, we inspected the change in CFI (ΔCFI) to evaluate the invariance, with values ≥ 0.010 indicating non-invariance, as (Chen, 2007).

## 3 Results

### 3.1 Measurement invariance over time and across subgroups for overall injustice

The results suggest strict measurement invariance for overall justice over time (CFI = 0.926, TLI = 0.921, SRMR = 0.052). Also, the change in CFI compared to the strong invariance model indicated strict invariance across subgroups (CFI = 0.901, TLI = 0.906, SRMR = 0.064, ΔCFI<0.01). A detailed presentation of the fit statistics for all models is given in Tables A1 and A2 in the Appendix.

### 3.2 Descriptive statistics

Workplace locked-in employees had higher overall injustice over time compared to those not being locked-in (Table 1). More specifically, the mean overall injustice varied from 3.47 in 2018 to 3.26 in 2020 for locked-in employees compared to 2.56 in 2018 and 2.41 in 2020 for those who were not locked-in, on Likert scale from 1 to 7. Workplace locked-in employees also reported both more frequent (28.25% in 2018; 28.16% in 2020) and longer (23.66% in 2018; 26.96% in 2020) sickness absence than those not locked-in (frequent sickness absence: 21.56% in 2018; 21.23% in 2020; long sickness absence: 20.93% in 2018; 23.93% in 2020). The correlation between frequency and duration of sickness absence was around 0.5 (p < 0.001) in either wave, indicating a rather moderate association between them.

**Table 1:** Descriptive statistics of the sample (n = 2631).

	2018		*P* value^a^	2020		*P* value
	Not locked-in	Locked-in		Not locked-in	Locked-in	
**Gender**			0.003			0.003
Women % (N)	60.49(1398)	48.13(154)		60.49(1398)	48.13(154)	
Men % (N)	39.51(913)	51.88(166)		39.51(913)	51.88(166)	
**Age groups, years** % (N)			0.285			0.208
<35	2.86 (66)	2.50 (8)		1.47 (34)	0.63(2)	
35-44	13.20 (305)	12.50 (40)		10.47(242)	12.19(39)	
45-54	35.83 (828)	41.88 (134)		30.85 (713)	35.63 (114)	
55-64	46.08(1065)	41.88(134)		50.11 (1158)	45.00 (144)	
65+	2.03 (47)	1.25(4)		7.10 (164)	6.56(21)	
**Socio-economic status** % (N)			<.001			<.001
Unskilled Workers	8.90 (200)	16.08 (50)		8.78 (201)	17.52 (55)	
Skilled Workers	16.15(363)	10.93(34)		16.07 (368)	10.19 (32)	
Assistant non-manual workers	10.54(237)	15.76(49)		10.87 (249)	16.56 (52)	
Intermediate non-manual workers	40.35(907)	30.23(94)		39.00 (893)	29.30 (92)	
Professionals, high civil servants	24.07(541)	27.01(84)		25.28(579)	26.43(83)	
**Overall injustice (mean, s.d.)**	2.56 (1.09)	3.47 (1.24)	<.001	2.41(1.08)	3.26 (1.28)	<.001
**Frequency of sickness absence (%, N)**			*0.008*			*0.005*
≥2 times	21.56 (492)	28.25 (89)		21.23 (485)	28.16 (89)	
<2 times	78.44 (1790)	71.75 (226)		78.77 (1800)	71.84 (227)	
**Duration of sickness absence (%, N)**			*0.266*			*0.237*
≥8 days	20.93 (480)	23.66 (75)		23.93 (548)	26.96 (86)	
<8 days	79.07 (1813)	76.34 (242)		76.07 (1742)	73.04 (233)	

a Chi2 test for categorical variables and ANOVA for continuous variables.

### 3.3 Multigroup SEM analysis

The results of SEM analysis are presented below.

### 3.4 Associations between overall injustice and frequency of sickness absence

**Fig. 2:**
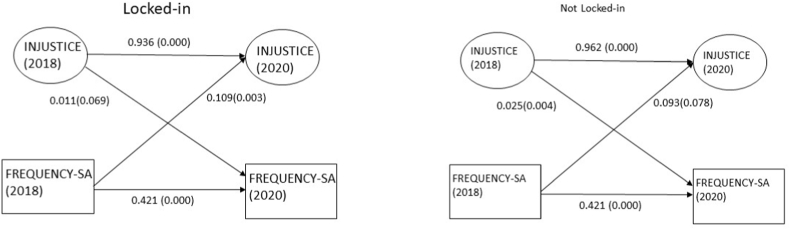
Multigroup SEM model of the relationship between overall injustice (INJUST) and frequency of sickness absence (FREQ_SA) in SLOSH 2018–2020 (n = 2631). The analyses were adjusted for gender, age, SEI, and change of boss. Fit statistics: RMSEA 0.074, CFI 0.886, TLI 0.871, SRMR 0.041.

Fig. 2 illustrates the estimated structural model between overall injustice and frequency of sickness absence stratified by locked-in status. For those who were not workplace locked-in, we found a statistically significant association between overall injustice in 2018 and frequent sickness absence in 2020 (beta = 0.025; *p* = 0.004). For those who were locked-in instead a reverse association was found: frequent sickness absence in 2018 significantly predicted overall injustice in 2020 (beta = 0.109; *p* = 0.003).

### 3.5 Associations between overall injustice and duration of sickness absence

**Fig. 3:**
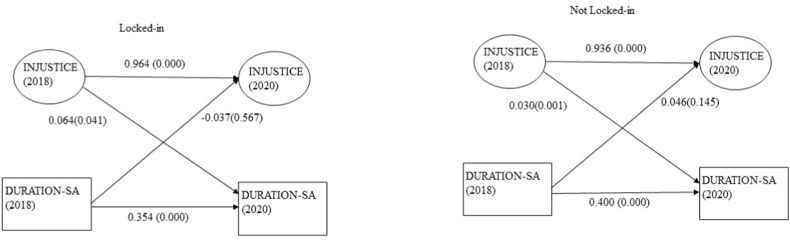
Multigroup SEM model of the relationship between overall injustice (INJUST) and duration of sickness absence (DUR_SA) in SLOSH 2018–2020 (n = 2631). The analyses were adjusted for gender, age, SEI, and change of boss. Fit statistics: RMSEA 0.074, CFI 0.886, TLI 0.870, SRMR 0.042.

The estimated paths between overall injustice and duration of sickness absence by locked-in status are presented in Fig. 3. Overall injustice in 2018 was significantly associated with long sickness absence in 2020, both for employees not being workplace locked-in (beta = 0.030; *p* = 0.001) and employees being workplace locked-in (beta = 0.064; *p* = 0.041). The path coefficient's difference was statistically significant (*p* < 0.001), indicating a stronger association between injustice and sickness absence among those who are workplace locked-in. When examining the reverse paths, the estimated coefficients were: beta = −0.037(*p* = 0.567) for the locked-in group and beta = 0.046 (*p* = 0.145) for the not-locked-in group but these were not statistically significant.

## 4 Discussion

In this study among individuals on permanent contracts in Sweden, we examined whether employees' workplace locked-in status modifies the association between overall injustice and subsequent sickness absence as defined through two differing measures of sickness absence. Frequent sickness absence provided a measure independently of absence length but does not allow any conclusions about the specific sickness absence spells’ length. On the other hand, long sickness absence indicated a need for a doctor certification when taking sick leave for more than seven days.

In line with what we expected, we found a positive association between organizational injustice and frequent sickness absence among those not beinglocked-in at the workplace. Also, higher injustice related positively to reporting taking long sickness absences (more than 7 days), independently of workplace locked-in status. However, the association was stronger in the locked-in group. Below the results are discussed in more detail.

Higher overall injustice at work was related to more frequent subsequent sickness absence for employees who were not locked-in. Employees who are not locked-in (Stengård, 2018) while reporting being treated unfairly may be better equipped to endure workplace adversity, due to having the possibility to leave the organisation for a better, less unfair, job. Indeed, the overall lower values of organisational injustice among not locked-in employees may confirm this. Another explanation is that employees treated unfairly at their workplace might make use of short but frequent sick leave, that do not require a doctor's certificate, to save energy and recover from stress. Individuals will offset the imbalance between what they put into work and what they get out by decreasing their work engagement, i.e., taking sickness absence more often. In this perspective, shorter, but frequent sick leave could be considered a coping mechanism (the ‘withdrawal’ explanation) as it might help an individual to get relief from high strain (Colquitt, 2001; Leineweber et al., 2017).

Interestingly, the association between overall injustice at work and frequent subsequent sickness absence did not reach statistical significance for employees being workplace locked-in. This may be partly be explained by the small size of the group and the lack of statistical power. Instead, workplace locked-in employees may decide to be present at work despite feeling disengaged or ill due to being afraid of losing their (non-preferred) job and lacking any other alternative potential employment. This is consistent with previous research suggesting that the fear of unemployment may force employees to work while being ill (Caverley et al., 2007; Hansen & Andersen, 2008; Heponiemi et al., 2010; Virtanen et al., 2005). Another reason that locked-in employees might not take sick leave is that they cannot afford to lose out on income due to sickness absence. Indeed a previous study found that locked-in individuals are most likely to have lower socio-economic status (Stengård et al., 2019).

When examining the duration of sickness absence, we found that higher overall injustice was related to long sickness absence both among workplace locked-in and not workplace locked-in employees. The association was stronger for those in workplace locked-in and the difference in the estimates between the two groups was statistically significant. The higher magnitude of the estimate for those in locked-in status suggests that overall injustice has a stronger influence on the length of sickness absence for this group of employees compared to those not feeling locked-in. If workplace locked-in employees avoid taking short-term sick leave, as previously suggested, then they may end up taking longer sick absences. This finding is also in line with previous research that has shown more workplace locked-in people having considerably worse health (Aronsson et al., 2019; Fahlén et al., 2009; Stengård et al., 2019).

The fact that overall injustice predicts long sickness absence for both groups of employees might be a sign of more serious health problems arising from prolonged stress due to poor work conditions (the “stress” explanation). Still the association was found to be stronger among those being workplace locked-in.

Finally, we found a reverse association for employees being locked-in, showing that frequent sickness absence is positively related to overall injustice two years later. This may imply that employees being locked-in more often take one day or two of short leave which in turn may increase their injustice perceptions.

### 4.1 Strengths and limitations

This study is among the first examining differences in the association between overall injustice and sickness absence by employees locked-in status. Using an overall measure of justice rather than specific types of justice has been recommended in previous research in order to avoid weakness related to the focus of specific types of justice (Ambrose & Schminke, 2009; Shapiro, 2001). In addition, this study extends previous literature, that has examined associations between injustice at work and sickness absence (Furåker & Saloniemi, 2014) by testing simultaneously differences in the two groups, being locked-in vs not being locked-in, through multigroup SEM models. This method has several advantages over running separate analyses for each group, such as providing more efficient parameters and allowing a test of significance of any differences found between groups. At the same time have these cross-lagged panel model been criticized for not disentangling between-person and within-person effects as well as not accounting for the trait-like time invariant nature of constructs (Mulder & Hamaker, 2021). However, in our data neither justice nor sickness absence are stable or trait like. In addition, although justice is quite stable, we can still predict it and there is evidence that justice changes. Another strength of our study is our measure of locked-in status. In order to construct the categorization of locked-in status, we combined employees’ workplace preferences and employability, which has been acknowledged to be a superior measure (Bernhard-Oettel et al., 2018) compared to measuring either preferences (Aronsson & Göransson, 1999) or employability (Furåker & Saloniemi, 2014). Finally, we examined different measures of sickness absence, which allows us to investigate the impact of overall injustice to frequent sickness absence (which could indicate a coping strategy with a stressful work environment) but also to long sickness absence related to more serious ill health problems.

Nonetheless, our study has also some limitations. First, we incorporated those being in risk zone and those being workplace locked-in in one group, in order to increase the sample size of the group. However, with more data at hand, a modified categorization of locked-in status, including a third group that captures employees “being at risk for locked-in”, i.e., those with (a) low preferences to remain in the workplace even if they were satisfied for the time being and (b) low employability, may be preferable (Stengård et al., 2016). Second, we included only permanent employees, since it has been discussed that being locked-in presumably has a different meaning for temporary and permanent workers (Stengård et al., 2016), such as the locked-in situation is terminated as soon as the contract ends. Third, by selecting only individuals in permanent employment, we have dropped all individuals that transitioned from or to temporary employment and unemployment as well as those retiring, which may imply the risk for a selection bias effect, particularly in relation to career changes. More research could identify the role of changes in employment status. Fourth, we do not know if the two years lag between waves in the SLOSH study is an optimal time frame to study associations between overall injustice and sickness absence. However, given that ill-health requiring sick leave make take some time to develop, a shorter time period would probably be insufficient. Also, our measures of sick leave cover the past twelve months, i.e., half of the follow-up time is covered. Fifth, we considered individuals being on frequent sickness absence if they had taken sick leave twice or more during the past 12 months. Choosing a stricter cut-off would have resulted in very small numbers in relation to the locked-in status, since when examining the distribution of sickness absence variables in relation to locked-in status, only about five percent of the participants reported to be on SA four times or more a year. Still, despite our rather ‘weak’ cut-off for sickness absence, we found that for those not workplace locked-in overall injustice in 2018 associated with frequency of sickness absence in 2020. Future work should examine alternative cut-offs may affect the results. Sixth, we found sickness absence to be less stable over time (predictive of itself) than organizational justice. Although, this might be surprising, justice perceptions are expected to change when changing one's employer. By excluding anyone who has changed their workplace or organization, we took away many of the reasons for organizational justice to change. Despite this, the results do show that sickness absence can predict justice perceptions and consequently there is at least some variance left in justice perceptions at Time 2 even when having taken into account justice perceptions at Time 1.

Finally, in the current study we cannot draw any conclusions about the impact of the Covid-19 pandemic, since the data collection for the year 2020 started in April 2020 when the pandemic was at its beginning. Our questions on organizational justice do not specify any time span and we believe that questions were mostly answered with thought on the ‘normal’, pre-pandemic, circumstances. However, we cannot exclude that sickness absence was influenced by the pandemic. However, as Table 1 shows, there is no increase in frequency of SA between 2018 and 2020 and only a slight increase in days of SA between the years. Future work, using pandemic data should examine the impact of the Covid-19 pandemic in the studied associations. Overall, future research should address these limitations in order to be able to fully understand the associations between overall injustice at workplace and sickness absence and the moderating role of workplace locked-in.

## 5 Conclusion

The present study found that overall injustice at work is related to frequent sickness absence for employees not being locked-in and to long sickness absence for all employees, independently of the locked-in status. This means that lack of justice at work can be a risk factor for as well frequent as long sickness absence both for employees being locked-in and those not being locked-in. Sickness absence is a concerning problem and related to individual suffering and income penalties, but also decreased productivity for the organization. The findings of the study also suggest that frequent sick leave is, for employees not being locked-in, potentially used as a coping technique to get relief from high strain while long sickness absence for both groups of employees might be a sign of more serious health problems due to a prolonged stress reaction to poor work conditions. In addition, we showed that feelings of being locked-in may intensify the negative and more serious health effects of overall injustice, which indicates that overall injustice for employees being locked-in is not only a risk factor for sickness absence but also for delayed return to work. Organizations should consider measures to improve employability and increase justice at work of workers in order to reduce long-term sick leave.

## Declaration of competing interest

The authors have no competing interests to declare.

## Figures and Tables

**Table 1:** Measurement invariance models for overall injustice among groups

Overall Injustice										
Model		χ^2^(df)	RMSEA	SRMR	CFI	TLI	Model Comparisons		Δχ^2^(p)	ΔCFI
							Δmodel	Δdf		
1	Configural invariance model	1287.43(114)	0.115	0.060	0.905	0.89				
2	Metric invariance model	1288.878(116)	0.114	0.06	0.905	0.892	2 vs 1	1	21.527(.000644)	0
3	Strong invariance model	1299.777 (121)	0.112	0.063	0.904	0.895	3 vs 2	5	5.852(.320887)	−0.001
4	Strict invariance model	1352.502 (139)	0.106	0.064	0.901	0.906	4 vs 3	18	29.722(.000044)	−0.003

**Table 2:** Measurement invariance models for overall injustice over time

Overall Injustice										
Model		χ^2^(df)	CFI	TLI	SRMR	RMSEA	Model Comparisons		Δχ^2^(p)	ΔCFI
							Δmodel	Δdf		
1	Configural invariance model	2087.400(52)	0.926	0.907	0.050	0.112				
2	Metric invariance model	2108.927(57)	0.926	0.914	0.052	0.107	2 vs 1	5	21.527(.000644)	0
3	Strong invariance model	2114.779(62)	0.926	0.921	0.052	0.103	3 vs 2	5	5.852(.320887)	0
4	Strict invariance model	2144.500(68)	0.925	0.927	0.052	0.099	4 vs 3	6	29.722(.000044)	−0.001

## Data Availability

The data that has been used is confidential.
